# Monitoring of Blood Clozapine Levels After a Change in Smoking Status for Patients Treated With Clozapine: A Clinical Audit From Hull Community Mental Health Team

**DOI:** 10.1192/bjo.2024.600

**Published:** 2024-08-01

**Authors:** Kamran Mahmood, Odira Anakebe, Keshia Theobalds-Ward, Madhu Gulati

**Affiliations:** Humber Teaching NHS Trust, Hull, United Kingdom

## Abstract

**Aims:**

According to The Medicines and Healthcare products Regulatory Agency (MHRA) Drug safety update in August 2020 regarding clozapine, monitoring blood clozapine levels for toxicity is now advised in certain clinical situations such as when a patient stops smoking or changes to e-cigarette.

Aim of this audit was to determine whether blood clozapine levels are being performed in patients on clozapine when there has been a change in patient’s smoking status from two localities, East and West Hull community mental health team.

Because there is a risk of significant blood clozapine change within 3–5 days post starting or stopping smoking which consequently increases the risk of toxicity, we also looked at whether a medical review was undertaken post change in smoking status in order to review if any adjustment was required in current clozapine dose.

**Methods:**

A list of Hull CMHT patients on clozapine was obtained from local clozapine clinic. The data comprised patients who were on clozapine from both localities of CMHT between October 2022 to October 2023.

Data was obtained retrospectively from Trust's patient electronic record system.

Eligibility criteria was set for the patient on clozapine to be a current smoker, or have been a smoker over last 12 months. Non-smokers and the ones on clozapine without a change in smoking status over the duration period were excluded.

58 patients were identified to be smokers and taking clozapine. Change in smoking status was documented in 21 instances, and therefore included in final analysis of results.

**Results:**

42.86% patients had a clozapine blood level check post smoking status change.

19% of patients from our sample had a medical review after change in smoking status within the duration time of audit.

**Conclusion:**

We concluded that compliance with current MHRA guidelines in relation to blood clozapine levels and change in smoking status is quite poor in Hull CMHT and measures are needed for improvement.

We recommend that every patient with a change in smoking status must have blood clozapine level checked within a week of any change in smoking status and a medical review in two weeks. We identified some scope of improving current clozapine monitoring form on electronic system and recommend changes by adding a section where change in smoking status is recorded.

